# A Molecularly Imprinted Polymer with Incorporated Graphene Oxide for Electrochemical Determination of Quercetin

**DOI:** 10.3390/s130505493

**Published:** 2013-04-25

**Authors:** Si Sun, Mengqi Zhang, Yijun Li, Xiwen He

**Affiliations:** College of Chemistry, Nankai University, 94 Weijin Road, Tianjin 300071, China; E-Mails: markfriendly51@gmail.com (S.S.); amengxiangxiao@126.com (M.Z.); xiwenhe@nankai.edu.cn (X.H.)

**Keywords:** molecularly imprinted polymer, graphene oxide, quercetin, modified electrode

## Abstract

The molecularly imprinted polymer based on polypyrrole film with incorporated graphene oxide was fabricated and used for electrochemical determination of quercetin. The electrochemical behavior of quercetin on the modified electrode was studied in detail using differential pulse voltammetry. The oxidation peak current of quercetin in B-R buffer solution (pH = 3.5) at the modified electrode was regressed with the concentration in the range from 6.0 × 10^−7^ to 1.5 × 10^−5^ mol/L (r^2^ = 0.997) with a detection limit of 4.8 × 10^−8^ mol/L (S/N = 3). This electrode showed good stability and reproducibility. In the above mentioned range, rutin or morin which has similar structures and at the same concentration as quercetin did not interfere with the determination of quercetin. The applicability of the method for complex matrix analysis was also evaluated.

## 1. Introduction

**Figure 1.**
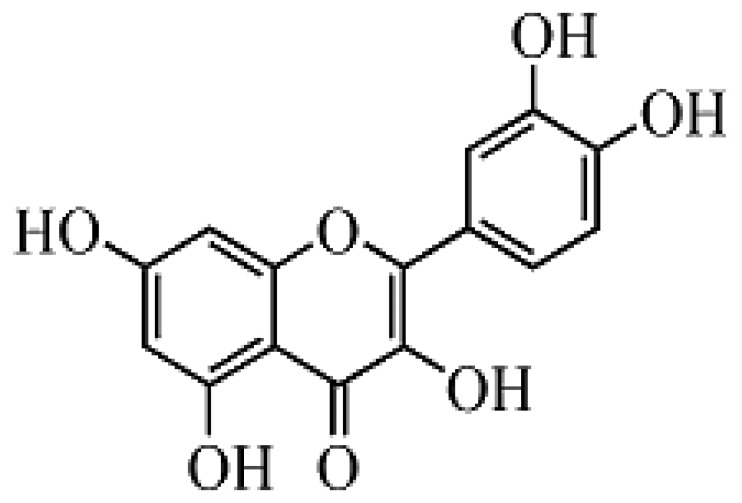
Structure of quercetin.

Flavonoids are natural products widely distributed in the plant kingdom and generally present in the common human diet. Evidence has shown that flavonoids are capable of modulating the activity of enzymes and affecting the behavior of many cell systems [1], and they are believed to be responsible for important health-enhancing effects [2]. Up to 2000, over 130 preparations containing certain kinds of flavonoids were registered as drugs worldwide [3]. Quercetin is the major representative of flavonol, one of the seven groups in the flavonoids family, a powerful antioxidant, and is regularly consumed by humans in edible fruits and vegetables at levels of up to 16 mg per day [4,5]. The molecular structure of quercetin is shown in Figure 1. Apart from flavonoids' therapeutic effects, quercetin is also excellent an scavenger of free radicals [6] which benefits humans immensely, therefore much attention has been paid to both the biological and physicochemical properties of quercetin over the past few decades. Hitherto, various analytical methods for the separation and determination of quercetin in flavonoids mixtures and real samples have been reported. High-performance liquid chromatography [7] and capillary electrophoresis [8–11] have been effectively used for the determination and quantification, coupled with various detection techniques [12], such as UV spectrophotometry, UV photodiode array detection, mass spectrometry, electrochemical detection and chemiluminescence. The above methods and their coupled techniques may provide high assay selectivity, but also leads to some disadvantages such as operational complexity, time and reagent consumption, high cost, *etc.* In fact, some direct methods for analysis of quercetin without pre-separation have also been described. They are mostly based on chemical derivitization combined with UV spectrophotometry [13], or a Kalman filter approach [14], but have a poor detection limit.

Because quercetin is electrochemically active, electrochemical methods with their advantages of higher sensitivity, low cost and less interferences from non-electroactive substances are preferable. The electrochemical oxidation of quercetin was studied by Nematollahi *et al.* [15] and Zare *et al.* [16] under different conditions. Conventional electrochemical methods with simple modification of the electrode surface can enable the determination of quercetin, despite the fact that strong absorption of quercetin on electrode surface leads to passivation of electrodes that hinders subsequent determination. Volikakis *et al.* [17] developed a way to detect quercetin on carbon paste electrodes through adsorptive stripping voltammetry. He *et al.* [18] utilized both the graphite-nujol paste electrode (GPE) and the carbon nanotubes-nujol paste electrode (CNTPE) for flavonoid determination. Recently, multiwall carbon nanotubes-modified electrodes have been found to be excellent electrodes for the determination of quercetin at trace levels due to their strong surface adsorption [19–21]. Electrodes modified with metal or metallic compounds are also used in quercetin detection [22–24].

The molecular imprinting technique (MIT) is becoming more and more commonly accepted as a useful method for recognition and isolation of key biological target molecules. When combined with template molecules, monomers may form multiple interactions, which are recorded through polymerization. After removal of template molecules, cavities come into being among the polymers which allow them to distinguish template molecules through their stereoconfiguration. Till now, it has been widely used in chromatographic [25], solid phase extraction [26], membrane separation [27], biological sensors [28] and electrochemical sensors [29,30], *etc*. Combined with MIT, the preparation of molecularly imprinted polymers (MIPs) in electrochemical sensors or biological sensors have considerable application prospects and important research significance [31,32]. Recently, there have been several studies on quercetin detection using MIT. Yu *et al.* [33] improved the selectivity of the quercetin MIP at low concentration. Pakade *et al.* [34] prepared quercetin MIPs in aqueous environment for selective recovery of quercetin from aqueous solutions at high temperature.

MIT has a great effect on improving the selectivity, but sensitivity is also an important factor for modified electrodes with excellent properties. It is proven that carbon-nanotubes and metallic nanoparticles contribute as well. Graphene now represents the new rising star in the field of material science and has attracted considerable attention in recent years. Structurally, graphene is a monolayer of tightly packed carbon atoms with a two-dimensional planar sheet of sp^2^-bonded carbon atoms arranged in a hexagonal configuration [35,36]. Since its experimental discovery in 2004, graphene has captured the interest of researchers because of its excellent properties, such as large surface-to-volume ratio, high conductivity and electron mobility at room temperature, robust mechanical properties, flexibility [37], good chemical stability, high transparency, and a broad electrochemical window [38]. Compared to tubular carbon nanotubes (CNTs), graphene oxide (GO) sheets could easily stick to glass carbon electrodes and fall off less easily. GO has a larger surface to volume ratio, a larger specific surface area, a greater atomic efficient utilization, and thus can improve electron transfer rates significantly. The unique structure and outstanding properties of graphene hold great promise for its applications, including photonic devices and photoluminescence [39], energy storage [40], photovoltaic devices [41,42], electrochemical supercapacitors [43] DNA-hybridization devices and drug delivery systems [44]. Because of their unique structural and electrical properties, graphene and graphene based materials can be fabricated as rapid and sensitive electrochemical sensors and nanodevices for small molecule determination, deoxyribonucleic acid (DNA) hybridization and protein–protein interactions [37,44]. In 2009, graphene was synthesized chemically by the Hummers and Offeman method and the graphene-modified electrode was first applied in selective determination of dopamine in a large excess of ascorbic acid [45]. From then on, graphene and grapheme-based materials have played important roles in the fabrication of chemical sensors as electrode modifiers. Combined with GO, several MIPs have been made into biochemical sensors [46,47]. Compared with other ways of synthesizing GO-MIP, electropolymerization methods have several advantages such as high fabrication rates, low material consumption and controllable film thickness, *etc*.

In this study, a method for determination of quercetin on a MIT/graphene modified glass carbon electrode was described. As far as we know, few paper about determination of quercetin using MIT on electrodes was reported [48,49]. In addition, this is the first time that GO was incorporated into a molecularly imprinted polymer for quercetin determination.

## 2. Experimental

### 2.1. Instruments and Materials

Electrochemical measurements were carried out on a model LK98B II microcomputer-based electrochemical analyzer (Lanlike Chemical High-tech Electronics Co. Ltd, Tianjin, China). A conventional three-electrode system was employed, including a working electrode, a platinum wire counter electrode and a saturated calomel electrode (SCE) reference electrode. Graphene oxide (purity >99%) was purchased from Vanguard Nanomaterial Reagent Co. Ltd. (Nanjing, China). Quercetin dehydrate, pyrrole, rutin trihydrate (Alfa Aesar, Tianjin, China), morin (Sigma-Aldrich, Shanghai, China) were of analytical grade and were used without further treatment. All aqueous solutions were prepared with reverse osmosis water (18.25 MΩ·cm).

### 2.2. Fabrication of Molecularly Imprinted Polymer Incorporated Graphene Oxide-Modified Electrode with Polypyrrole (MIP/GO/GC)

Glass carbon electrodes (GC, Φ = 4 mm) were successively polished with 0.05 μm aluminum oxide slurries on chamois leather until a mirror-like surface was obtained. After rinsing with water in each polishing step, the electrodes were subject to ultra-sonication in water-ethanol mixture (1:1 v/v). Cyclic voltammetry(CV) has been employed to test the performance of the electrode under the following conditions: 1.0 × 10^−3^ mol/L K_3_Fe(CN)_6_, 0.1 mol/L phosphate buffer solution (pH = 7.0), with potential rate of 0.6 V to 0.2 V and a scan rate of 0.05 V/s. graphene oxide (GO, 2 mg) was dispersed into DMF (50 mL) by ultrasonic agitation for 10 min to give a homogeneous suspension. A certain portion of the GO-DMF suspension was transferred onto a clean GC surface and the suspension was dried under an infrared lamp. A GO/GC was thus fabricated. Electropolymerization in the three-electrode system in a solution which contains 0.05 mol/L pyrrole, 0.01 mol/L quercetin and 0.1 mol/L H_2_SO_4_ on GO/GC was carried out using chronocoulometry at 0.7 V for 100 s. Cyclic voltammetric experiments were performed on the modified electrode in 0.1 mol/L PBS (pH = 7.0) in a potential range from −0.2 to 1.3 V with a scan rate of 0.10 V/s for the over oxidation of polypyrrole film. Non-molecularly imprinted polymer electrode (NIP/GO/GC) was prepared as above, but without the template.

### 2.3. Electrochemical Determination of Quercetin with MIP/GO/GC

The working electrode was dipped into water-ethanol mixture (1:1 v/v) containing quercetin for a period of time for adsorption and then transferred to Britton-Robinson (B-R) buffer solution. Differential pulse voltammetry (DPV) experiments were performed in 0.1 mol/L B-R buffer solution (pH = 3.5). Experimental parameters were optimized according to the voltammetric response of quercetin in the solution.

## 3. Results and Discussion

### 3.1. Preparation of MIP/GO/GC

**Figure 2.**
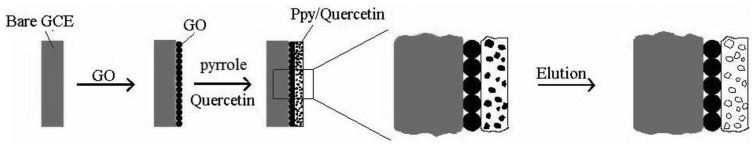
Fabrication process of the MIP/GO/GC.

A MIP/GO/GC was fabricated as shown in Figure 2. Template molecules bound with functional monomer through hydrogen bonds. Under cyclic voltammetric scanning in a large range, hydrogen bonds were broken by potential change, therefore template molecules lose bonding force and were eluted. Electrochemical elution was preferable with its advantages of rapid response, good reproducibility, and furthermore resulted in the film's long-term stability.

**Figure 3.**
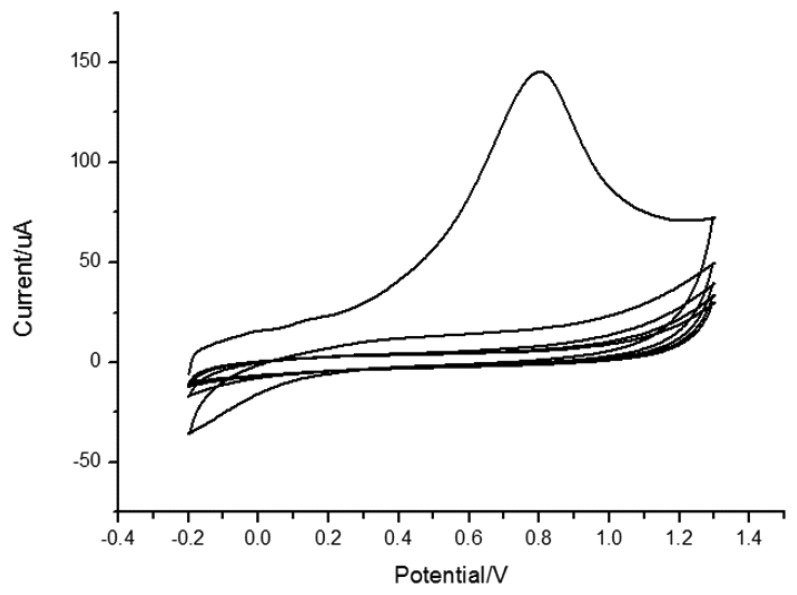
CV of over oxidation of polypyrrole film and quercetin elution on MIP/GO/GC in 0.1 mol/L PBS (pH = 7.0). Scan rate: 0.1 V/s.

In 0.1 mol/L PBS (pH = 7.0), the electrochemical behavior of MIP/GO/GC was studied by using cyclic voltammetry in a scan range of −0.2 V and 1.3 V as shown in Figure 3. Compared with the first and the second cycle, the anodic peak at 0.8 V was disappeared at the second cycle, which indicated polypyrrole (PPy) was over oxidized.

Shiigi *et al* reported that the overoxidation of polypyrrole could improve the selectivity of molecularly imprinted film [50]. The polypyrrole skeleton will lose electroactivity after overoxidation under neutral or alkaline conditions while oxygen containing groups, such as carboxyl, are introduced into the skeleton. Under these conditions, template molecules are eluted from the polypyrrole film and thus a molecularly imprinted electrode is fabricated. Moreover, the anionic template is entrapped within the polymer during electropolymerization in the imprinting scheme based on overoxidized PPy. Subsequent overoxidation replaces the conventional washing step and creates nanocavities with template expulsion [31]. At the same time, a small peak belonging to quercetin at 0.2 V also vanished, which demonstrated that quercetin was eluted completely. The cyclic curves of the 4th and 5th cycle almost coincided with each other, which proved that the activation was complete.

### 3.2. Influence of Different GO contents

Different amounts of GO dispersed suspension were dropped onto the GC surface. After the electrode surface was dried, MIP/GO/GC and NIP/GO/GC were fabricated through electro-polymerization and overoxidation. Subsequently, MIP/GO/GC was eluted and activated, while NIP/GO/GC was directly activated. MIP/GO/GC and NIP/GO/GC containing different amounts of graphene were put into water-ethanol mixture (1:1 v/v) containing 5.0 × 10^−6^ mol/L quercetin for adsorption for 5 min. After rinsing with water, differential pulse voltammetry (DPV) method was used to determine quercetin in 0.1 mol/L Britton-Robinson (B-R) buffer solution (pH = 3.5). The electrochemical results are summarized in Table 1.

From the above table, we can see that the response current of MIP and NIP increased with increasing content of graphene because of the accelerated electron transfer rate of graphene. However, thick graphene layers would block electrons from reaching the electrode surface which leads to a current decrease at high graphene content. Based on the results listed in Table 1, 5.0 μL (0.2 μg) GO suspension was dropped on GC for the preparation of the electrodes.

### 3.3. Influence of Supporting Electrolyte and pH

**Figure 4.**
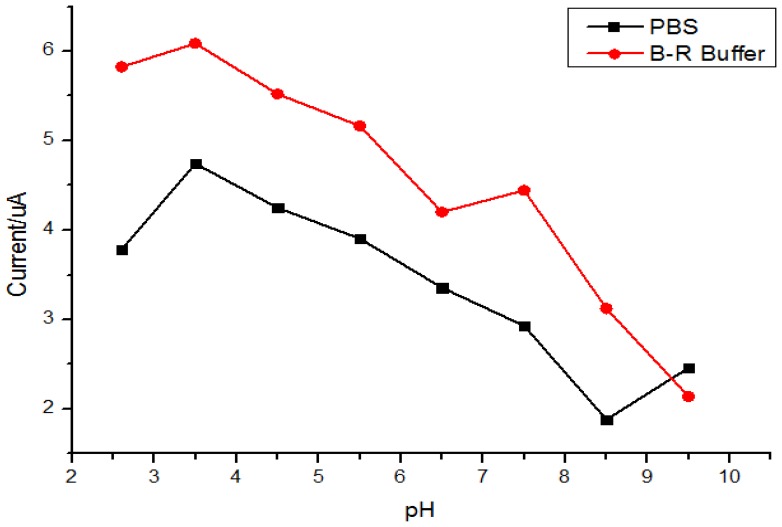
Influence of buffer solution pH by PBS or B-R buffer.

A series of buffer solutions with different pH values were prepared from 0.1 mol/L B-R or PBS solutions. MIP/GO/GC was put into water-ethanol mixture (1:1 v/v) containing 5.0 × 10^−6^ mol/L quercetin for adsorption for 5 min. After rinsing with water differential pulse voltammetry (DPV) experiments were carried out in the respective buffer solutions and the results are shown in Figure 4. The results indicated that a higher peak current and better peak shape could be obtained in a B-R buffer solution (pH = 3.5). This was attributed to the boric acid in the B-R buffer solution. Being electron-deficient atoms, boron atoms could attack the quercetin molecule effectively by electrostatic interactions with the hydroxyl group of quercetin. Moreover, poor response at high pH values could be due to deprotonation of polypyrrole [51].

### 3.4. Influence of Adsorption Time

**Figure 5.**
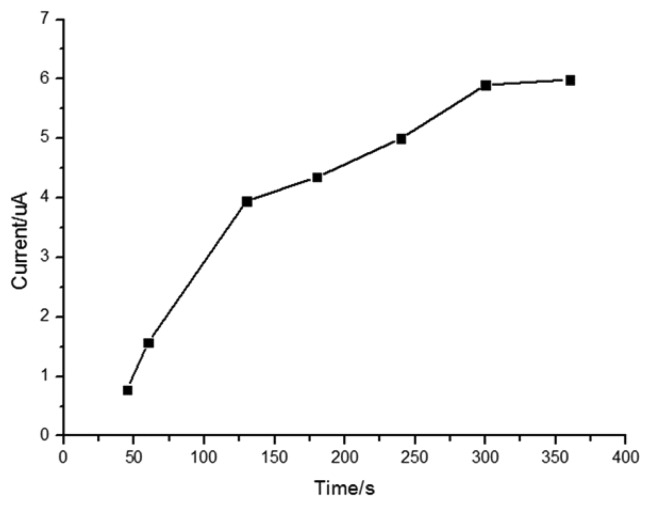
Variation of peak current with adsorption time.

It has been reported that MIP films can reach absorption saturation in a short period of time [52,53]. Figure 5 shows that the peak current increases with adsorption time at the beginning of our experiments, but after five minutes, the peak current reaches a plateau value because of adsorption saturation, so the adsorption period of five minutes was selected for determination of quercetin.

### 3.5. Calibration Curve

**Figure 6.**
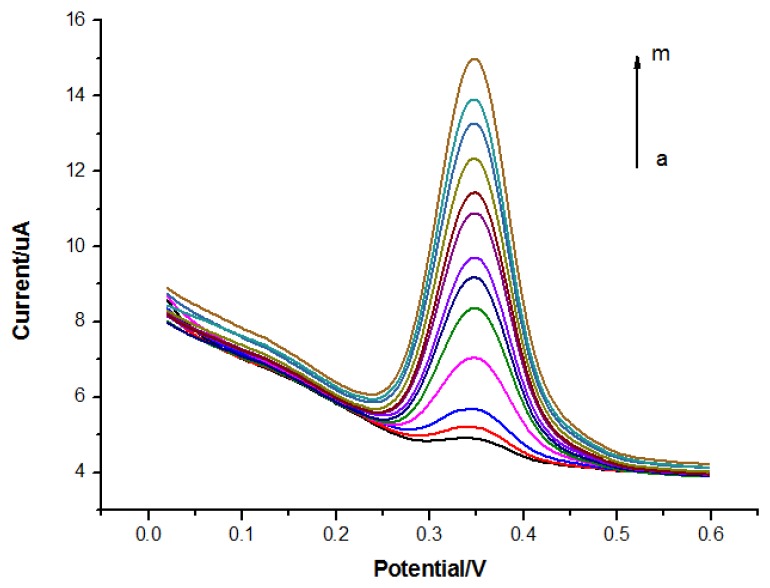
Differential pulse voltammetry on the concentrations of quercetin in the range from 6.0 × 10^−7^ to 1.5 × 10^−5^ mol/L (a∼m).

**Figure 7.**
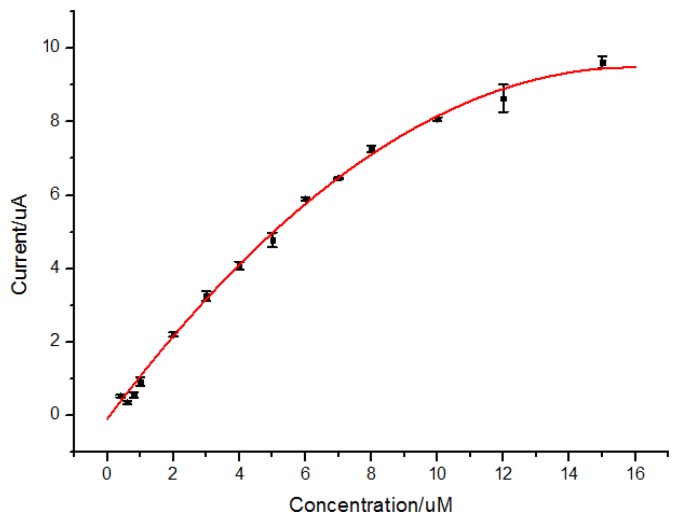
Relationship between peak current and quercetin concentration.

The DPV responses of quercetin in solutions in different concentrations were recorded as shown in Figure 6. The oxidation peak current of quercetin was dependent on the concentration of quercetin in the range from 6.0 × 10^−7^ to 1.5 × 10^−5^ mol/L (r^2^ = 0.997) by the equation of I(μA) = (−0.04 ± 0.003)C^2^(μmol^2^/L^2^) + (1.20 ± 0.033)C(μmol/L) + (−0.09 ± 0.075), with a detection limit of 4.8 × 10^−8^ mol/L (S/N = 3) as shown in Figure 7. The non-linear response of the electrodes may be attributed to both specific absorption and nonspecific absorption on the MIP/GO/GC electrodes.

### 3.6. Stability and Repeatability of MIP/GO/GC

Under the optimal conditions mentioned above, a 6.0 × 10^−6^ mol/L quercetin solution was measured successively for seven times with the same electrode. The relative standard deviation (RSD) of the peak current was 1.2%. Then the electrode was stored in water for 15 days. Afterwards, a 6.0 × 10^−6^ mol/L quercetin solution was investigated by the electrode under same experimental conditions for more than 300 scanning segments. The peak current decreased no more than 5%. In addition, five different MIP/GO/GC electrodes were prepared and tested in a 6.0 × 10^−6^ mol/L quercetin solution and the RSD of the peak current was 1.8%. The results indicated that MIP/GO/GC had good stability and repeatability.

### 3.7. Interferences

**Figure 8.**
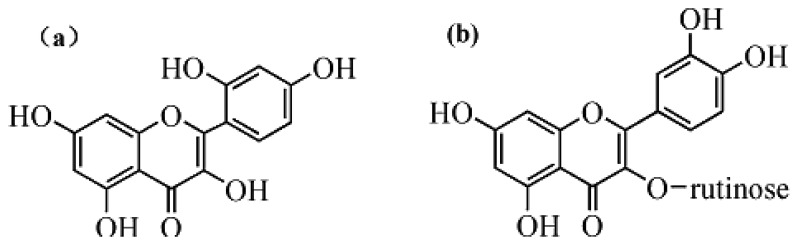
Structures of (**a**) morin and (**b**) rutin.

**Figure 9.**
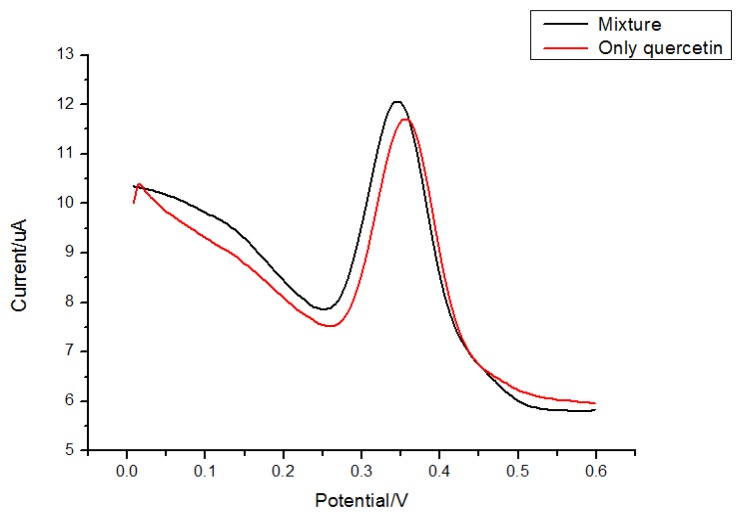
DPV of 5.0 × 10^−6^ mol/L quercetin in a mixture of morin of the same concentration.

**Figure 10.**
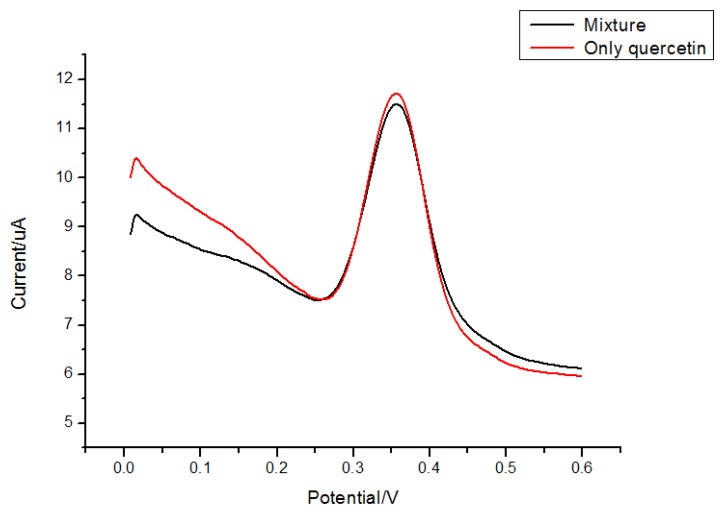
DPV of 5.0 × 10^−6^ mol/L quercetin in a mixture of rutin with the same concentration.

Rutin and morin (Figure 8) have similar structures as quercetin. Thus they were selected to test interferences in the proposed determination of quercetin. Considering the fact that the total contents of quercetin, rutin and morin are similar in most plants, quercetin solution was prepared by adding interferents with the same concentration, so a mixed solution of 5.0 × 10^−6^ mol/L morin and 5.0 × 10^−6^ mol/L quercetin was tested for the interference study. Results (Figures 9 and 10) showed that 5.0 × 10^−6^ mol/L morin or rutin would not affect the determination of quercetin at the same concentration. In this sense, the electrode proposed in this paper had a high selectivity.

### 3.8. Analysis of Quercetin in Complex Matrices

Fruit juices usually contain quercetin in the range from 2.96 × 10^−6^ to 3.84 × 10^−5^ mol/L. Newly expressed apple juice (10.0 mL) was used as a real sample and was diluted five times with water-ethanol mixture (1:1 v/v) before the determination. The quercetin concentrations in our samples were calculated using the standard addition method under optimized conditions. The results shown in Table 2, indicate that MIP/GO/GC could be used in the determination of quercetin in complex matrices with satisfactory results.

## 4. Conclusions

Graphene oxide has a large surface-to-volume ratio and high electron transmission rate which improves significantly the sensitivity of electrochemically modified electrodes based on graphene. MIT can increase the selectivity of modified electrode considerably. MIP/GO/GC could be obtained simply by electropolymerization of pyrrole in the presence of the template (quercetin) on graphene oxide modified electrode. MIP/GO/GC has a polynomial response in the range from 6.0 × 10^−7^ to 1.5 × 10^−5^ mol/L (r^2^ = 0.997) with a detection limit of 4.8 × 10^−8^ mol/L (S/N = 3). The selectivity of MIP/GO/GC was tested by using rutin and morin which have similar structures. The results showed that MIP/GO/GC had a good imprinting effect for the recognition of quercetin. The application of MIP/GO/GC was also tested by determining quercetin in newly expressed apple juice.

## Figures and Tables

**Table 1.** Influence of different GO contents on the determination of quercetin.

**Volume of GO Dispersed Suspension Dropped on the Electrode Surface(μL)**	**NIP/GO/GC (μA)**	**MIP (μA)**	**Imprinting Factor**
0.0	0.00	0.55	
2.0	0.79	0.89	1.13
3.0	0.72	1.52	2.11
4.0	0.84	2.47	2.94
5.0	0.79	4.48	5.67
5.5	0.89	2.25	2.53
6.0	0.86	1.97	2.29

**Table 2.** Determination of quercetin in apple juice with MIP/GO/GC by using the standard addition method.

**Addition (μmol/L)**	**Measured (μmol/L)**	**Recovery (%)**	**RSD (n=3) (%)**
0.000	0.759	-	-
1.994	2.791	101.4	1.40
2.989	3.689	98.4	1.60
3.485	4.239	99.9	0.52
3.981	4.619	97.4	1.29
4.476	5.260	100.5	2.89
